# The Impact of Dietary Berberine Supplementation during the Transition Period on Blood Parameters, Antioxidant Indicators and Fatty Acids Profile in Colostrum and Milk of Dairy Goats

**DOI:** 10.3390/vetsci9020076

**Published:** 2022-02-11

**Authors:** Navid Ghavipanje, Mohammad Hasan Fathi Nasri, Seyyed Homayoun Farhangfar, Seyyed Ehsan Ghiasi, Einar Vargas-Bello-Pérez

**Affiliations:** 1 Department of Animal Science, Faculty of Agriculture, University of Birjand, Birjand 97175-331, Iran; hfathi@birjand.ac.ir (M.H.F.N.); hfarhangfar@birjand.ac.ir (S.H.F.); s.e.ghiasi@birjand.ac.ir (S.E.G.); 2 Department of Veterinary and Animal Sciences, Faculty of Health and Medical Sciences, University of Copenhagen, DK-1870 Frederiksberg C, Denmark

**Keywords:** antioxidants, berberine, fatty acid profile, inflammation, transition goat

## Abstract

The objective of this study was to investigate the effect of berberine (BBR) supplementation on productivity, antioxidant markers, and the fatty acid (FA) profile in the colostrum and milk of goats. Twenty-four primiparous Saanen goats were supplemented with 0, 1, 2, and 4 g/d (per goat) of BBR in control (CON), BBR1, BBR2, and BBR4 groups (*n* = 6 per group), respectively, from 21 days before expected kidding to 21 days after parturition. Blood sampling was carried out at −21, −14, −7, 0, 7, 14, and 21 d relative to delivery. Colostrum was collected within the first and second milking (d 1 of lactation), and milk was harvested weekly after kidding. Both BBR2 and BBR4 increased dry matter intake (DMI) (*p* ≤ 0.05) and energy balance (EB) as well as colostrum and milk production. Both BBR2 and BBR4 decreased (*p* ≤ 0.05) plasma levels of cholesterol, haptoglobin, and ceruloplasmin, while elevating the plasma albumin and paraoxonase (*p* ≤ 0.05), which may indicate that BBR mitigates inflammation during the transition period. BBR reduced (*p* ≤ 0.05) malondialdehyde (MDA) and increased (*p* ≤ 0.05) total antioxidant capacity (TAC), superoxide dismutase (SOD), glutathione peroxidase (GSH-Px), and catalase (CAT) in blood, colostrum, and milk. Concentrations of de novo fatty acid in colostrum and milk were increased (*p* ≤ 0.05) with both BBR2 and BBR4. Free fatty acid (FFA) concentration in colostrum and milk fat were lower (*p* ≤ 0.05) in BBR2 and BBR4 compared to CON. The concentration of saturated fatty acids (SFAs) in colostrum and milk fat increased (*p* ≤ 0.05) with BBR2 and BBR4, while unsaturated fatty acids (USFAs) decreased (*p* ≤ 0.05) in milk. In summary, supplementation with at least 2 g/d BBR may enhance the EB and antioxidant status of dairy goats.

## 1. Introduction

The perinatal dairy goat, from approximately 21 d prepartum to 21 d postpartum, is commonly defined as the transition goat [1,2]. Transition goats are metabolically challenged during that period [2,3]. Apart from negative energy balance (NEB) caused by high energy requirements at the beginning of lactation and an inadequate feed intake [2,3], goats during the transition period can also show elevated plasma free fatty acid concentration [2], low-grade inflammation [2,3], and oxidative stress [1], similar to cows [4,5,6,7].

Moreover, throughout the transition period, dairy goats can have significant alterations of metabolic status, pro-oxidant/antioxidant status, and in their immune systems [1,8]. This oxidative stress is caused by, among other things, enhanced metabolic activity in consequence of the increased energy demands, reduced antioxidant capacity, and increased reactive oxygen species (ROS) production [8,9].

Research has shown that NEB, along with increased circulated non-esterified fatty acids (NEFA) and β-hydroxybutyrate (BHBA), induces immunosuppression and increases the release of positive acute phase proteins, such as haptoglobin and ceruloplasmin in transition cows [4,10]; however, limited information is available for dairy goats during the transition period [1]. Also, alterations in a dairy animal’s metabolic homeostasis might change the milk component and FA profile [11]. It is well established [12,13] in dairy cows that not only milk fat content can be affected but also single milk FA, which can also be used as indicators of an animal’s metabolic status. Studies have verified that the milk composition and FA profile are influenced by the lactation stage [14] and energy status [15] in dairy goats; however, there is still little known about indicators in milk for assessing the metabolic status of early-lactation goats.

Berberine (5,6-dihydrodibenzo [a,g] quinolizinium), a natural isoquinoline alkaloid from the protoberberine class, is originated from different herbal families such as the Berberidacae, Ranunculaceae, and Papaveraceae families [16]. The main sources of BBR are the rhizomes, roots, and stem bark of barberry (*Berberis vulgaris*), goldenseal (*Hydrastis canaden*sis), coptis (*Coptis chinensis*), tree turmeric (*Berberis aristata*), and Oregon grape (*Berberis aquifolium*) [17,18]. During the last few decades, many studies have shown that BBR exerts various beneficial effects [16,17,18,19], such as lowering blood glucose, regulating blood lipids, encouraging antioxidant activity, reducing inflammation, and increasing insulin sensitivity, thus ameliorating insulin resistance (IR) [16,17,18,19]. In addition, the therapeutic benefits of BBR in treating type 2 diabetes mellitus and metabolic syndromes have been reviewed previously [16,17,18,19,20]. BBR inhibits oxidative stress and inflammation in a variety of tissues, including liver, adipose, kidney, and pancreas [19].

To the best of our knowledge, scarce information is available with regard to NEB, oxidative stress, and inflammation in transition goats as well as on health status and milk production. This article is part of a larger study that evaluated the effects of BBR supplementing on performance, glucose metabolism, insulin sensitivity, inflammation, and oxidative stress status in periparturient dairy goats. Our companion paper [21] revealed that BBR ameliorates the NEB in transition goats. The objective of the preset study was to reassess BBR supplementation as a novel preventive approach towards boosting antioxidant indicators in blood and milk, as well as its effects on FA profile in the colostrum and milk of goats during the transition period.

## 2. Materials and Methods

This study was carried out on an experimental farm at the University of Birjand, located in Birjand, Iran (longitude and latitude, 37.42° N and 57.31° E). The research protocol and animal care were in accordance with guidelines of the Iranian Council of Animal Care [22] on the protection of animals used for scientific purposes.

### 2.1. Design, Animal, and Diet

The trial was carried out with 24 Saanen dairy goats during the last 21 days of pregnancy and the first 21 days of lactation. Goats were housed in individual stalls (1.8 m × 1.6 m). Pre- and post-partum diets were formulated to be isocaloric [2.60 and 2.90 Mcal metabolizable energy (ME) dry matter (DM) basis in pre- and post-partum diets, respectively] and isonitrogenous [18.5 and 15.5% crude protein (CP) DM basis in pre- and post-partum diets, respectively] in order to fulfill the requirements of the national research council (NRC) [23] (Table 1). Goats were fed a total mixed ration (TMR) with a 45:55 forage/concentrate ratio, twice daily in equal amounts (06:00 and 14:00 h) for ad libitum consumption and had free access to water.

Amounts of feed offered and refused were recorded daily. Following parturition and through 21 d of lactation, milking was performed daily using a portable milking machine (Tim Gibson Ltd., Bedale, UK) at 05:30 and 15:30 h. Goats were grouped in a completely randomized design, and treatments were supplementation of 0, 1, 2, and 4 g/d of BBR for each goat in control (CON), BBR1, BBR2, and BBR4 groups (*n* = 6 per group), respectively. These dosages corresponded to 0 to 100 mg dietary BBR per kg body weight (BW). Berberine hydrochloride (BBR HCL) (BulkSupplements, Eastgate, Henderson, NV, USA) was put in gelatin capsules (Irancapsul, Tehran, Iran) and orally administrated to each goat with a balling gun (Pars Khavar, Tehran, Iran) before morning feeding from 21 days prior to anticipated kidding date through 21 days of lactation. To the best of our knowledge, there is no comparable data regarding the effects of BBR in cattle; however, the levels of BBR were chosen based on previous in vitro [18] and in vivo [16,17,18,19,20] experiments.

### 2.2. Blood Collection and Analysis

Blood (10 mL/goat) was collected from each animal after overnight fasting, once weekly from 21 days before expected kidding, up to 21 days of lactation. Blood was harvested from the jugular vein using heparinized blood collection tubes (RotexMedica, Trittau, Germany). Blood samples were centrifuged at 3000× *g* at room temperature for 15 min. Plasma was obtained and stored at −80 °C until analyzed. Plasma samples were analyzed for concentrations of albumin, cholesterol, glucose, and bilirubin using commercial kits (Pars Azmun Co., Ltd., Tehran, Iran) by an autoanalyzer (BT 1500, Biotecnica SpA, Rome, Italy). Plasma haptoglobin and ceruloplasmin were determined using commercial test kits (Randox Laboratories Ltd., Ardmore, UK). Plasma paraoxonase (PON) activity was measured by adapting the method of Ferre´ et al. [24]. Serum insulin level was measured using enzyme linked immunosorbent assay kit (Monobind Inc., Lake Forest, CA, USA). In addition, BHBA and NEFA were measured using commercial kits (Randox Laboratories Ltd., Ardmore, UK).

### 2.3. Colostrum and Milk Collection and Analysis

Colostrum yield per individual goat was determined as the sum of the amounts obtained from the first and second milkings (day 1 after kidding), and colostrum samples (50 mL/goat) were taken. Goats were milked twice daily at 05:30 and 15:30 h, and milk yield was recorded after each milking. Weekly milk samples were collected from 2 consecutive milking times following parturition. Colostrum and milk samples were preserved with potassium bichromate and stored at 4 °C until analyzed. Samples for milk fatty acid (FA) and antioxidant concentrations did not have chemical preservation. Milk fat, true protein, lactose, total solids, and solid non-fat content were determined using a Fourier transform mid-infrared (FTIR) spectrophotometer (Lactoscope FTA, Delta Instruments, Drachten, the Netherlands) at the Rojan Alvand certified laboratory (Karaj, Iran). The prediction models used were the optimized basic model of filtered wavelengths and intercorrection factors described by Kaylegian et al. [25]. The reference methods for fat, protein, and lactose measurement were determined in duplicate using the following validated methods [26]: fat by modified Mojonnier ether extraction (method 989.05), true protein by Kjeldahl analysis (method 991.22), and lactose by enzymatic analysis (method 2006.06).

De novo and preformed milk FA were estimated by FTIR using the partial least squares (PLS) prediction models described by Woolpert et al. [27]. Gas-liquid chromatography reference chemistry for calibration of milk FA parameters was used, as described by Wojciechowski and Barbano [28].

### 2.4. Antioxidants Assays in Blood Plasma, Colostrum and Milk

The level of malondialdehyde (MDA) in plasma, colostrum, and milk was determined using the thiobarbituric acid method according to Placer et al. [29]. The results were obtained as nmol/mL using the colorimetric method. Total antioxidant capacity (TAC) in the colostrum and milk was determined by the ferric-reducing antioxidant power (FRAP) method [30] with some modifications according to Smet et al. [31]. Before measuring absorbance, a centrifugation step was introduced (1300× *g* for 5 min), resulting in a clear medium. Plasma TAC was measured using a Randox kit (Crumlin, County Antrim, UK) in which 2.2-azino-bis (3-ethylbenzothiazoline-6-sulfanate, ABTS) was incubated with a peroxidase and H_2_O_2_ to produce the radical cation ABTS^+^. This had a stable blue-green color, which is measured at 600 nm. The enzymatic activities of superoxide dismutase (SOD), glutathione peroxidase (GSH-Px), and catalase (CAT) in either blood or colostrum/milk were measured using the commercial Randox kit (Randox Laboratories Ltd., Ardmore, UK) according to the manufacturers’ procedures.

### 2.5. BCS, Energy Calculation, and Other Measurements

For all goats, body condition score (BCS) was determined on days −21, −14, −7, 0, 7, 14, and 21, relative to parturition. The same technician assessed BCS for each doe on a scale of 1 (emaciated) to 5 (over-conditioned) as described by Villaquiran et al. [32].

Pre- and post-partum energy balance (EB) was calculated according to NRC [23]:

EB = NEC − NEM − NEG − NEP(1)

NEC = NEL/kg of DM × DMI(2)

NEM = BW0.75 × 0.08 × 1.1(3)

NEG = (0.00318 × days of gestation − 0.0352) × (kid birth weight/0.45)/0.218(4)

NEP = milk, kg × [(0.0929 × fat, %) + (0.0547 × protein, %) + (0.0395 × lactose, %)](5)

where, NEC = net energy consumed, NEL = net energy of lactation, NEM = net energy of maintenance, NEG = net energy requirement of pregnancy, and NEP = net energy output in milk. In addition, metabolizable protein intake (MPI) was obtained from the following formula [23]:

MPI (g/d) = DMI (kg) × MP (g/kg DMI)(6)

### 2.6. Data Analysis

Collected data were analyzed in a completely randomized design using the MIXED procedure of Statistical Analysis System 9.2 software [33]. The fixed effects in the model were: the dietary treatment (BBR supplementation), the time of sampling (Time), and their interaction (Diet × Time), while individual goats were included as a random factor. Three covariance structures were tested (autoregressive-1, spatial power, and unstructured), and the one resulting in the lowest Akaike information criterion was chosen. The REPEATED procedure was used for all variables. Least square means (LSM) separation between time points was performed using the PDIFF statement by Tukey’s test and presented as LSM ± SEM. Significance was declared at *p* ≤ 0.05 and trends at *p* ≤ 0.10 and *p* ≥ 0.05.

## 3. Results

### 3.1. DMI, BCS, and EB

Supplementation of either BBR2 or BBR4 increased (*p* ≤ 0.05) the DMI of goats in the overall transition period as compared to the control group (Table 2). Moreover, intakes of NEL and MP were higher (*p* ≤ 0.05) for goats receiving supplemental BBR2 or BBR4. The energy balance values as Mcal/d or % requirements of goats were increased (*p* ≤ 0.05) by BBR supplementation, followed by BBR2 and BBR4. Goats supplemented with BBR tended (*p* = 0.10) to lose less BCS than unsupplemented goats. However, BW (*p* ≥ 0.05) was similar between treatments.

### 3.2. Biomarkers of Inflammation and Health Status

Changes of health status biomarkers in periparturient dairy goats are shown in Table 3. Compared with the CON group, biomarkers of health status were improved with BBR feeding. Supplementation with both BBR2 and BBR4 increased (*p* ≤ 0.05) plasma albumin and paraoxonase concentrations. Data showed that either BBR2 or BBR4 treatment reduced (*p* ≤ 0.05) plasma concentrations of cholesterol, haptoglobin, and ceruloplasmin, upon transition period. Also, bilirubin tended (*p* = 0.09) to decrease with increasing BBR supplementation. No significant effect (*p* ≥ 0.05) was observed on plasma glucose, whereas, supplementation of BBR2 or BBR4 increased (*p* ≤ 0.05) insulin concentration of transition does. Moreover, BBR2 and BBR4 reduced (*p* ≤ 0.05) the NEFA concentration in transition period, and a tendency (*p* = 0.10) was observed for decreased BHBA in goats fed BBR compared with CON.

### 3.3. Colostrum and Milk Production and Components

Colostrum and milk production are shown in Table 4. Supplementary BBR did not affect (*p* ≥ 0.05) colostrum and milk components. The highest (*p* ≤ 0.05) colostrum yield was observed in BBR2 group, followed by BBR4. Increased (*p* ≤ 0.05) milk yield was observed in both BBR2- and BBR4-fed goats.

### 3.4. Colostrum, Milk, and Blood Antioxidant Status

Milk and blood TAC in the does supplemented with BBR2 and BBR4 was higher than in CON and BBR1 groups (Table 5, *p* ≤ 0.05). Indeed, colostrum TAC represented the highest (*p* ≤ 0.05) value in BBR4, followed by BBR2 and BBR1. Likewise, BBR2- and BBR4-supplemented goats had lower (*p* ≤ 0.05) MDA concentration in colostrum and blood. The lowest (*p* ≤ 0.05) MDA in milk was observed in BBR2, followed by BBR4 and BBR1. In terms of enzymatic antioxidant, the activity of SOD in blood, colostrum, and milk were higher (*p* ≤ 0.05) in does supplemented with either BBR2 or BBR4. GSH-Px in colostrum and blood were increased (*p* ≤ 0.05) in BBR supplemented goats compared with control. Likewise, the GSH-Px activity in milk was higher (*p* ≤ 0.05) in BBR2 and BBR4 groups. The CAT activity in colostrum and blood of does supplemented with either BBR2 or BBR4 decreased (*p* ≤ 0.05) compared with the corresponding values of CON group. Similarly, the milk CAT activity increased (*p* ≤ 0.05) with either BBR2 or BBR4 supplementation during the transition period.

### 3.5. Colostrum and Milk Fatty Acid Profile

The goats’ colostrum and milk FA profile are presented in Table 6. Supplementation of BBR2 and BBR4 increased concentrations of de novo FA in colostrum and milk (*p* ≤ 0.05). Supplementation of BBR to transition goats reduced performed FA in colostrum (*p* = 0.02) and milk (*p* = 0.04) fat in BBR4, followed by BBR2 as compared to the CON diet. FFA concentration in colostrum and milk fat was decreased in goats receiving either BBR2 or BBR4 compared to the CON diet (*p* ≤ 0.05). The concentration of SFAs in colostrum and milk fat were increased (*p* ≤ 0.05) with BBR2 and BBR4, while USFAs were decreased in milk (*p* ≤ 0.05). In the present study, although the concentration of MUFAs in colostrum was reduced with BBR2 and BBR4 supplementation, PUFAs were not affected in either colostrum or milk (*p* ≥ 0.05). The content of C18:0 in colostrum increased (*p* ≤ 0.05) compared with the CON diet when BBR2 and BBR4 were supplemented for transition goats. In addition, both BBR2 and BBR4 decreased (*p* ≤ 0.05) the percentage of C18:1n9 (oleic acid) in colostrum, with the greatest decline being for the BBR2 group.

## 4. Discussion

The transition period imposes tremendous stress on dairy goats and is recognized as a critical period because of anatomical, physiological, biochemical, and hormonal alterations that lead to metabolic, immune, and antioxidant imbalance, which can result in inflammation [1,3] and oxidative stress [8,9], and may result in inadequate nutrient and energy intake to support the onset of lactation [1,2,3], similar to dairy cows [4,5,7,10]. In this study, we determined whether BBR supplementation would modulate the colostrum and milk production of transition Saanen goats, as well as their antioxidant status. Not only are there scarce data available on performance, milk, and colostrum antioxidants and adaptive response indices of transition goats [1], but also, no investigation has evaluated the BBR effect on dairy ruminants, especially transition goats. Hence, to the best of our knowledge, this is the first study exploring changes in colostrum and milk antioxidants and FA profile of transition dairy goats.

### 4.1. Dry Matter Intake, Body Condition Score, and Energy Balance

Transition from late gestation to early lactation is a stressful period that is faced by the vast majority of dairy animals with NEB [3]. It is well-established that dairy goats are nutritionally challenged in this phase, with increasing energy requirements and the limitation of voluntary feed intake that make periparturient goats vulnerable to metabolic diseases [1,2,3]. Thus, the need for finding alternatives to mitigate NEB and improve performance has great importance for transition dairy animals [34]. With regard to performance, DMI, BW, and BCS were in line with earlier findings from transition dairy goats [3,35]. In this study, we found that supplementing 2 or 4 g BBR/d (BBR2 and BBR 4, respectively), increased DMI, as well as the intake of NEL, MP, and EB in transition goats. Lima et al. [36] reported that hormone-induced adaptations in transition cattle lead to reduction in DMI and serious imbalances in energy status. In addition, a positive correlation between DMI and intakes of NEL and MP, as well as EB, has been reported in goats [36] and cows [37]. Thus, our data suggest potential involvement of BBR supplementation in improving DMI and EB via the inhibition of excess body fat mobilization in periparturient goats.

In agreement with current findings, our recent report using the same animal cohort [21] revealed that BBR ingestion in transition goats enhanced pre- and post-partum intakes of DM and NEL. Likewise, it has been reported that BBR enhances energy homeostasis via AMP-activated protein kinase (AMPK) activation [16,38,39,40]. AMPK, an AMP-dependent protein kinase, is a critical molecule in the regulation of bioenergetic metabolism and metabolic-related diseases [16,40]. In line with our study, numerous reports [16,40,41] retrieved from both in vivo and ex vivo studies have shown that BBR can improve energy homeostasis.

Collard et al. [42] reported that a decrease in BCS is evidence of tissue mobilization in severe cases of NEB in transition cows; similarly, a recent study [3] verified BCS as an indicator of body fat reserve usage. De Vries and Veerkamp [43] reported that BCS losses are correlated with fat mobilization, and therefore, BCS might be used as indicator for EB during the transition period. Thus, in the present study, the tendency to increase BCS of both BBR2 and BBR4 supplemented goats is another confirmation of enhanced energy homeostasis in these groups. Taken together, the use of BBR in transition goats may prevent NEB.

### 4.2. Biomarkers of Inflammation and Health Status

It is well-established that NEB and particular indicators of lipolysis, such as NEFA and BHB, are implicated in immuno-suppressive and inflammation-modulating effects [1,4]. Similar to that of cows [5], the NEB in transition goats is characterized by lower blood glucose levels and elevated blood concentrations of NEFA and BHBA [3], which is consistent with the present study. Interestingly, supplementation of either BBR2 or BBR4 (i.e., 2 and 4 g BBR per day) increased plasma albumin and paraoxonase concentrations, while reducing fat mobilization indicators (NEFA and BHBA) and inflammation biomarkers (haptoglobin and ceruloplasmin), which demonstrated the potential alleviative effect of BBR on fat metabolism disorder and inflammation initiated by NEB in periparturient goats.

In this study, plasma from BBR-supplemented goats had an increased concentration of negative APP and decreased levels of the positive APP, which agrees with in vitro studies in mouse neuroblastoma N18TG2 cell line [44] and in human neuroblastoma SH-SY5Y cells [45]. Li et al. [19] have demonstrated the anti-inflammatory effect of BBR associated with alleviation of the APP and proinflammatory cytokines. Similarly, Wang et al. [17] reported that BBR inhibited inflammation by regulating different signaling pathways and cytokines. In addition, the expression of pro-inflammatory genes in the adipose tissue in obese mice was inhibited by BBR, which indicated that BBR moderates both the acute and low-grade inflammatory responses [38]. One possible mechanism for the anti-inflammatory effect of BBR may be through activated protein kinase activation. Jeong et al. [38] reported that BBR represses pro-inflammatory responses through activated protein kinase activation in macrophages and significantly down-regulates the expression of pro-inflammatory genes. Further studies should consider analyzing the expression of genes related to inflammation when BBR is supplemented in transition dairy goats.

Serum values of glucose, insulin, and NEFA from this study indicated the occurrence of body fat mobilization in transition dairy goats. The BBR administration increased serum insulin concentrations and reduced NEFA and BHBA in transition goats, which implies its potential capacity for ameliorating fat mobilization. Similar to these results, Zamuner et al. [46] reported that reduction in NEFA and BHBA concentrations, as well as accretion in insulin and glucose, indicated increased body reserve usage and NEB. In this study, the decreases of plasma NEFA and the tendency of BHBA reflected the mitigated mobilization of body fat [4,5,46] in BBR-supplemented goats.

### 4.3. Colostrum and Milk Performance and Components

In the current study, the positive effect of BBR on colostrum and milk yields could be due to the higher DMI and EB in goats receiving the supplemental BBR [6,23]. Positive associations between transition energy status and subsequent production in goats have been reported in numerous studies [35,36]. In line with our results, van Knegsel et al. [47] showed that, when energy demands for milk production are fulfilled by feed intake, this leads to improved EB and reduced body fat mobilization, resultant in higher milk production.

The higher colostrum and milk yields could also be attributed to the anti-inflammatory role of the BBR since increments in indicators of inflammatory status, have consistently been linked to poor performance in dairy goats [1,8,9]; however, most data on this effect have been collected in dairy cows [4,5,6,7]. Indeed, the impact of metabolic inflammation on production has been shown, of which cows with higher levels of inflammatory markers had reduced milk production in the first month, compared to cows presenting lower levels of inflammation [10]. Farney et al. [48] showed that the anti-inflammatory treatment of transition cows increased milk yield by about 7%. All this shows that, since DMI is a major factor limiting colostrum and milk production in early lactation [1,4,5], enhanced DMI in goats that received supplemental BBR2 and BBR4 along with mitigated inflammation leads to higher intakes of NEL and MP, which results in better colostrum and milk yields of goats.

### 4.4. Colostrum, Milk, and Blood Antioxidant Status

Studies performed in the last decade clearly indicate that dairy goats [8,9] experience oxidative stress (OS) in the transition period, as do cows [7]. Colostrum and milk fat of goats is very susceptible to oxidation [49], which is a major deteriorative reaction that occurs during the processing, distribution, and storage of milk and dairy products, and, moreover, it can cause chemical changes that affect the nutritional quality, integrity, and safety of the milk [49,50]. In addition, given that colostrum is the first meal that a kid should receive shortly after birth, its antioxidant content is important to offsetting birth-associated OS [7,8,9]. The antioxidative properties of colostrum and milk could be attributed to the presence of enzymes, such as GSH-Px, SOD, and CAT [49,50,51]. There are many studies on the positive relationship between the blood and milk antioxidant status [49,52]. The current results showed that, compared with CON, plasma, colostrum, and milk TAC increased with BBR2 and BBR4. In addition, MDA concentration in either plasma or colostrum and milk decreased with BBR supplementation. This finding is similar to earlier findings [16,18,20] where BBR was reported to reduce free radicals and exert radical scavenging activity. Moghaddam et al. [20] reported that BBR significantly decreases serum MDA and increases TAC in alloxan-induced diabetic rats. In line with our findings, Malekinezhad et al. [53] showed that BBR supplementation restored the level of MDA in aflatoxin-B1- and ochratoxin-contaminated broilers. In addition, it has been reported that feeding goats with *Berberis vulgaris* leaf (a natural rich source of BBR) leads to greater TAC [54].

In the current study, the overall increase in enzymatic antioxidants of blood, colostrum, and milk with BBR supplementation in transition dairy goats, in particular BBR2 and BBR4, is in line with previous studies on the antioxidant effects of BBR supplementation. As such, the antioxidant effect of supplementation of 50 and 100 mg BBR/kg/day (the dose corresponding with BBR2 and BBR4, respectively) has been corroborated by studying its inhibitory effect on the up-regulation of glial fibrillary acidic protein (GFAP), a key indicator of astrogliosis which occurs as a result of oxidative stress in diabetic rats [20]. BBR enhanced the activity of antioxidant enzymes both in vitro and in vivo [17,20,53]. In addition, BBR significantly improved enzymatic antioxidants, such as SOD, CAT, and GSH-Px, in azoxymethane-induced carcinogenic rats [55]. Another study showed that BBR restores the activity of SOD in rats with type-1 diabetes mellitus [16]. Investigation on antioxidants, GSH-Px, and SOD enzymes concluded that BBR restored the levels of both SOD and GSH-Px, thereby reducing the elevated levels of lipid peroxidation in diabetic rats [41]. In the current study, the superior antioxidant properties of the blood, colostrum, and milk of BBR-supplemented goats suggest that BBR could prevent oxidative stress in transition goats.

### 4.5. Colostrum and Milk Fatty Acid Profile

As in cows [11,12,13], in dairy goats, milk FA are related to the stage of lactation, and that relates to energy balance [14]. Milk FA originate directly from the diet, from de novo synthesis in the mammary gland, from formation in the rumen by biohydrogenation or bacterial degradation, and from the release from body fat stores [56].

Negative energy balance leads to greater proportions of SFAs, mainly C16:0 and C18:0; therefore, using milk FA as an indicator for the metabolic status of lactating animals could be an indirect method for monitoring energy status [15,56]. This agrees with our results showing that supplementation with BBR2 and BBR4 increased the concentrations of de novo FA in colostrum and milk and had a tendency to increase mixed FA. Van Knegsel et al. [47] suggested that, during NEB, de novo synthesis of FA (C6:0 to C14:0) was reduced and body fat reserves were used. Hence, the promoted de novo milk FA in BBR-supplemented goats was probably due to their EB, which may lead to increased de novo FA synthesis in colostrum and milk as a result of less body fat reserve usage. Therefore, in the present study, the increased SCFA concentrations in both the colostrum and milk from the BBR2 and BBR4 groups are likely due to improved energy balance.

Likewise, the decline in de novo and mixed FA of the CON group indicated an increase in fat mobilization from reserves during early lactation, which was mitigated by BBR supplementation. This is in agreement with Bionaz et al. [56], who reported that the high uptake of long-chain FA by mammary gland tissue inhibits de novo synthesis of FA through the inhibition of acetyl-coenzyme a carboxylase. It has been shown [11] that about half of the mixed FA originates from de novo synthesis. Similarly, Woolpert et al. [27] reported an increase in mixed FA concentration following increases in de novo FA. Therefore, this could be a possible explanation for a tendency to increase mixed FA in BBR–fed goats.

In the current study, preformed FA concentrations (sum of FA > C16) were decreased in BBR2 and BBR4 as a result of enhanced EB, with a similar pattern described by Kay et al. [12] who reported that the preformed fatty acids were decreased with lactation progress and improvement in EB. Similarly, Gross et al. [11] declared that de novo and preformed FA reflected changes in the energy balance of dairy cows. In addition, Ducháček et al. [15] reported that Holstein cows in NEB had lower SFA and higher unsaturated fatty acid (UFA) milk contents, which agrees with our results, suggesting that goats with lower EB produced colostrum and milk with a higher content of UFA.

Strzałkowska et al. [14] reported that the FFA level is an indicator of EB in goat milk. A NEB occurring during the early stage of lactation leads to lipolysis of adipose tissue, which simultaneously increases FFA in goat´s milk [14]. Similarly, our results confirmed that improved EB in goats that received supplemental BBR was associated with a decrease in FFA. This also agrees with Stoop et al. [13], who reported that milk SFAs decrease, while MUFAs, mainly represented by C18:1 c9 decrease with improved energy balance.

In line with our results, Bionaz et al. [56] reported that excessive amounts of NEFA, released during body fat mobilization, are also transferred to milk, resulting in increased contents of milk long-chain FA, such as C18:1 c9 and C18:0. Hence, lower C18:1n-9 and C18:0 concentrations in colostrum and milk from either BBR2 or BBR4 might be linked to enhanced EB. It is well-established that oleic acid (C18:1 c9) is the predominant FA in adipocytes, which is primarily released through lipolysis during NEB [11,15]. Gross et al. [11] reported that an elevated proportion of C18:1 c9 in milk fat is a suitable biomarker for NEB. This was also confirmed by Gross et al. [11], who reported that in early-lactating cows with NEB, there is a decrease in short-chain FA and an increase in long-chain FAs. Moreover, increases of C18:1 c9 often occur at the expense of de novo FA in milk fat when cows increase their mobilization of body fat reserves [11,15]. In dairy goats, the increase in relative percentage of C18:1 c9 has been reported during periods of NEB [57].

## 5. Conclusions

In summary, supplementation with BBR during the transition period can increase DMI, EB, intakes of NEL and MP, and reduce plasma biochemical indicators of NEB and plasma biomarkers of inflammation (positive APPs) in dairy goats. Supplementation with BBR increased colostrum and milk yields, as well as improving antioxidant indicators in blood, colostrum, and milk, demonstrating its potential positive effect on productivity, immunity, and antioxidative capacity in transition dairy goats. Overall, our findings suggest that supplementation of 2 and/or 4 g/d BBR (i.e., 50 and/or 100 mg BBR per kg BW) may enhance EB and improve performance in transition dairy goats.

## Figures and Tables

**Table 1:** Ingredients, chemical analysis and nutritive value dry matter (DM) basis of the pre- and post-partum basal total mixed rations.

	Diets ^a^	
	Pre-Partum	Post-Partum
Ingredient (% of DM)		
Alfalfa hay	4.00	29.5
Corn silage	34.3	10.8
Wheat straw	17.9	5.00
Barley grain, ground	7.70	10.8
Corn grain, ground	31.5	22.2
Soybean meal	1.00	17.0
Wheat bran	1.80	2.20
Calcium–carbonate	0.90	1.00
Minerals and vitamins premix ^b^	0.90	0.50
Salt	0.00	1.00
Chemical composition		
Metabolizable energy, Mcal/kg of DM	2.60	2.90
Crude protein (% DM)	18.5	15.5
Ether extract (% DM)	2.50	2.50
Ash (% DM)	7.60	8.00
Natural detergent fiber (% DM)	43.0	37.3
Non-fibrous carbohydrates (% DM) ^c^	38.0	36.7

^a^ Pre-partum = from 50 days before parturition until kidding; post-partum = from kidding until 21 days of lactation. ^b^ Containing vitamin A [250,000 International Unit (IU)/kg], vitamin D (50,000 IU/kg), vitamin E (1500 IU/kg), manganese (2.25 g/kg), calcium (120 g/kg), zinc (7.7 g/kg), phosphorus (20 g/kg), magnesium (20.5 g/kg), sodium (186 g/kg), iron (1.25 g/kg), sulfur (3 g/kg), copper (1.25 g/kg), cobalt (14 mg/kg), iodine (56 mg/kg), and selenium (10 mg/kg). ^c^ Non-fibrous carbohydrates (NFC) were estimated according to the equation: NFC = 100 − (NDF + CP + EE + Ash) [23].

**Table 2:** Effect of Berberine (BBR) supplementation on intakes of dry matter (DMI), net energy for lactation (NEL), and metabolizable protein (MP) and body condition score (BCS) of transition dairy Saanen goats ^1^.

Item ^2^	Treat ^3^				SEM ^5^	*p* Values
	CON	BBR1	BBR2	BBR4		
DMI, Kg/d	1.58 ^b^	1.68 ^b^	1.97 ^a^	1.94 ^a^	0.040	<0.001
NEL intake, Mcal/d	1.99 ^b^	2.13 ^b^	2.50 ^a^	2.45 ^a^	0.054	<0.001
MP intake, g/d	208.37 ^b^	224.77 ^b^	264.09 ^a^	258.47 ^a^	6.152	<0.001
Energy balance, Mcal/d	0.58 ^c^	0.73 ^bc^	1.25 ^a^	1.22 ^ab^	0.121	0.004
Energy balance, %	123.46 ^b^	138.27 ^b^	182.63 ^a^	183.72 ^a^	6.670	<0.001
Body weight, Kg	46.12	46.92	47.26	46.51	1.511	0.854
BCS ^4^, point	3.31	3.34	3.48	3.47	0.0570	0.102

^a–c^ Within a row, means without a common uppercase superscript letter differ (*p* < 0.05). ^1^ Values are least-square means. ^2^ Data cover the last 21 days of gestation through the first 21 days of lactation. ^3^ CON = diet without BBR supplementation; BBR1, BBR2, BBR4 = diet supplemented with 1, 2, and 4 g/d of BBR, respectively. ^4^ Body condition score (BCS). ^5^ SEM = pooled standard error of mean.

**Table 3:** Effect of Berberine (BBR) supplementation on biomarkers of inflammation and health status in transition dairy Saanen goats ^1^.

Item ^2^	Treat ^3^				SEM ^4^	*p* Values
	CON	BBR1	BBR2	BBR4		
Inflammation						
Albumin, mg/dL	4.05 ^b^	4.22 ^b^	4.94 ^a^	4.76 ^a^	0.063	<0.001
Cholesterol, mg/dL	70.62 ^a^	68.61 ^ab^	64.38 ^b^	64.80 ^b^	1.433	0.041
Bilirubin, mg/dL	0.30	0.28	0.23	0.25	0.019	0.091
Haptoglobin, g/L	0.46 ^a^	0.40 ^a^	0.31 ^b^	0.29 ^b^	0.016	0.004
Ceruloplasmin, μmol/L	3.70 ^a^	3.60 ^ab^	3.30 ^b^	3.36 ^b^	0.078	0.018
Paraoxonase, U/mL	125.90 ^c^	126.71 ^bc^	132.19 ^a^	131.14 ^ab^	1.260	0.017
Energy balance						
Glucose, mg/dL	59.62	56.90	61.28	61.14	2.011	0.432
Insulin, μ/mL	19.40 ^b^	21.57 ^b^	26.27 ^a^	27.13 ^a^	0.905	0.001
Non-esterified fatty acids, mmol/L	0.16 ^a^	0.15 ^ab^	0.10 ^b^	0.11 ^b^	0.012	0.028
β-hydroxybutyrate, mmol/L	0.48	0.43	0.32	0.34	0.051	0.100

^a–c^ Within a row, means without a common uppercase superscript letter differ (*p* < 0.05). ^1^ Values are least-square means. ^2^ Data cover the last 21 days of gestation through the first 21 days of lactation. ^3^ CON = diet without BBR supplementation; BBR1, BBR2, BBR4 = diet supplemented with 1, 2, and 4 g/d of BBR, respectively. ^4^ SEM = pooled standard error of mean.

**Table 4:** Effect of Berberine (BBR) supplementation on colostrum and milk performance of dairy Saanen goats ^1^.

Item ^2^	Treat ^3^				SEM ^4^	*p* Values
	Control	BBR1	BBR2	BBR4		
Colostrum						
Yield, kg/first 2 milkings	0.80 ^b^	1.20 ^b^	1.62 ^a^	1.40 ^ab^	0.197	0.050
Protein, %	9.41	8.22	9.13	8.10	0.914	0.680
Fat, %	9.83	8.75	7.01	7.82	1.783	0.596
Lactose, %	4.01	4.15	4.10	4.19	0.123	0.743
Total solids, %	21.54	19.90	19.74	18.63	1.639	0.668
Solid non-fat, %	13.77	12.87	13.73	12.93	0.790	0.725
Milk						
Yield, kg/d	1.70 ^b^	1.98 ^ab^	2.54 ^a^	2.15 ^a^	0.155	0.013
Protein, %	4.56	3.92	3.76	3.63	0.363	0.314
Fat, %	4.96	4.74	4.55	4.15	0.582	0.695
Lactose, %	3.94	4.47	4.52	4.70	0.253	0.227
Total solids, %	14.01	13.89	12.16	12.82	0.598	0.101
Solid non-fat, %	9.28	9.27	8.61	9.31	0.196	0.074

^a–b^ Within a row, means without a common uppercase superscript letter differ (*p* < 0.05). ^1^ Values are least-square means. ^2^ Data cover the last 21 days of gestation through the first 21 days of lactation. ^3^ CON = diet without BBR supplementation; BBR1, BBR2, BBR4 = diet supplemented with 1, 2, and 4 g/d of BBR, respectively. ^4^ SEM = pooled standard error of mean.

**Table 5:** Effect of Berberine (BBR) supplementation on antioxidant status of colostrum, milk, and blood in transition dairy Saanen goats ^1^.

Item ^2,3^	Treat ^4^				SEM ^5^	*p* Values
	CON	BBR1	BBR2	BBR4		
Blood						
TAC, mmol/L	0.26 ^b^	0.28 ^b^	0.34 ^a^	0.34 ^a^	0.012	0.002
MDA, nmol/L	1.76 ^a^	1.60 ^a^	1.32 ^b^	1.34 ^b^	0.110	0.050
SOD, U/g Hb	1498.16 ^b^	1555.30 ^b^	1771.22 ^a^	1725.20 ^a^	47.301	0.009
GSH-Px, U/g Hb	39.95 ^b^	42.15 ^a^	46.68 ^a^	44.34 ^a^	1.421	0.048
CAT, U/g Hb	17.68 ^b^	21.34 ^ab^	25.94 ^a^	27.52 ^a^	2.350	0.050
Colostrum						
TAC, mmol/L	1.07 ^c^	1.15 ^bc^	1.42 ^ab^	1.51 ^a^	0.081	0.014
MDA, nmol/L	2.50 ^a^	2.27 ^ab^	1.90 ^b^	1.95 ^b^	0.134	0.044
SOD, U/mL	22.73 ^b^	24.4 ^ab^	26.9 ^a^	26.57 ^a^	0.814	0.021
GSH-Px, U/mL	59.34 ^b^	63.68 ^ab^	77.00 ^a^	73.00 ^a^	3.912	0.041
CAT, U/mL	15.75 ^b^	17.43 ^ab^	22.23 ^a^	21.08 ^a^	1.490	0.047
Milk						
TAC, mmol/L	1.26 ^b^	1.37 ^b^	1.67 ^a^	1.65 ^a^	0.064	0.003
MDA, nmol/L	3.24 ^a^	2.97 ^ab^	2.41 ^c^	2.52 ^bc^	0.122	0.004
SOD, U/mL	23.87 ^c^	25.31 ^bc^	29.01 ^a^	28.73 ^ab^	1.291	0.050
GSH-Px, U/mL	74.89 ^b^	83.78 ^b^	97.34 ^a^	82.17 ^a^	2.54	0.005
CAT, U/mL	20.17 ^b^	21.93 ^b^	27.35 ^a^	27.21 ^a^	1.342	0.011

^a–c^ Within a row, means without a common uppercase superscript letter differ (*p* < 0.05). ^1^ Values are least-square means. ^2^ Data cover the last 21 days of gestation through the first 21 days of lactation. ^3^ TAC = total antioxidant capacity, MDA = malondialdehyde, SOD = superoxide dismutase, GSH-Px = glutathione peroxidase, CAT = catalase. ^4^ CON = diet without BBR supplementation; BBR1, BBR2, BBR4 = diet supplemented with 1, 2, and 4 g/d of BBR, respectively. ^5^ SEM = pooled standard error of mean.

**Table 6:** Effect of Berberine (BBR) supplementation on colostrum and milk fatty acid profile of dairy Saanen goats ^1^.

Item ^2,3^	Treat ^4^				SEM ^5^	*p* Values
	CON	BBR1	BBR2	BBR4		
Colostrum						
De novo fatty acids, %	32.63 ^b^	32.50 ^b^	39.52 ^a^	38.22 ^a^	1.414	0.007
Mixed fatty acids, %	26.18	30.51	28.50	32.12	2.855	0.637
Preformed fatty acids, %	40.19 ^a^	36.77 ^ab^	31.90 ^b^	29.66 ^b^	2.244	0.025
FFA, meq/100g fat	15.37 ^a^	14.90 ^a^	10.35 ^b^	11.03 ^b^	0.826	0.001
SFA, %	61.70 ^b^	63.82 ^ab^	69.20 ^a^	69.55 ^a^	1.910	0.030
UFA, %	23.51	22.31	18.19	19.10	2.348	0.359
MUFA, %	14.30 ^a^	16.77 ^a^	8.44 ^b^	9.91 ^ab^	1.900	0.032
PUFA, %	6.73	5.53	5.74	5.14	0.501	0.283
C16:0, %	21.84	19.77	20.02	21.73	1.075	0.411
C18:0, %	7.72 ^a^	6.15 ^ab^	4.24 ^b^	3.57 ^b^	0.824	0.016
C18:1 c9, %	12.73 ^a^	14.93 ^a^	5.97 ^b^	7.96 ^ab^	1.890	0.020
Milk						
De novo fatty acids, %	38.02 ^a^	38.46 ^ab^	39.77 ^ab^	41.03 ^b^	0.831	0.050
Mixed fatty acids, %	34.09	40.08	38.19	38.60	1.646	0.114
Preformed fatty acids, %	27.88 ^a^	21.47 ^ab^	21.25 ^ab^	20.27 ^b^	1.857	0.049
FFA, meq/100g fat	10.85 ^a^	9.54 ^a^	6.51 ^b^	6.98 ^b^	0.540	0.003
SFA, %	64.97 ^b^	65.17 ^b^	69.65 ^a^	70.02 ^a^	1.01	0.045
UFA, %	21.84 ^a^	19.88 ^a^	18.28 ^ab^	17.29 ^b^	1.06	0.049
MUFA, %	14.54 ^a^	15.73 ^a^	12.61 ^b^	12.02 ^b^	0.623	0.004
PUFA, %	4.03	4.12	3.90	3.92	0.599	0.901
C16:0, %	29.38	28.83	29.56	29.19	0.940	0.796
C18:0, %	8.04	7.40	6.96	5.84	1.171	0.612
C18:1 c9, %	14.84	14.58	10.66	10.77	1.373	0.081

^a–b^ Within a row, means without a common uppercase superscript letter differ (*p* < 0.05). ^1^ Values are least-square means. ^2^ Data cover the first 21 d of lactation. ^3^ FFA = free fatty acids; SFA = saturated fatty acids; UFA = unsaturated fatty acids; MUFA = mono-unsaturated fatty acids; PUFA = poly-unsaturated fatty acids. ^4^ CON = diet without BBR supplementation; BBR1, BBR2, BBR4 = diet supplemented with 1, 2, and 4 g/d of BBR, respectively. ^5^ SEM = pooled standard error of mean.

## Data Availability

All of the required data have been presented in our article.
